# Adipokine gene expression in peripheral blood of adult and juvenile dermatomyositis patients and their relation to clinical parameters and disease activity measures

**DOI:** 10.1186/s12950-015-0075-2

**Published:** 2015-04-09

**Authors:** Jeannette M Olazagasti, Molly Hein, Cynthia S Crowson, Consuelo Lopez de Padilla, Erik Peterson, Emily C Baechler, Ann M Reed

**Affiliations:** Division of Rheumatology, Department of Medicine, Mayo Clinic, Rochester, MN USA; Department of Health Sciences Research, Mayo Clinic, Rochester, MN USA; Department of Medicine, Division of Rheumatic and Autoimmune Diseases, and Center for Immunology, University of Minnesota, Minneapolis, MN USA; Department of Pediatrics, Duke University School of Medicine, 201 Trent Drive, Durham, NC 27710 USA

**Keywords:** Dermatomyositis, Adipokines, Biomarkers, Visfatin, Resistin, Leptin, Adiponectin

## Abstract

**Objective:**

Recently adipokines have been implicated in the regulation of immune and inflammatory responses in autoimmune disease. To investigate the role of adipokines in adult and pediatric patients with newly diagnosed dermatomyositis (DM), we analyzed peripheral blood and skeletal muscle gene expression of four adipokines: visfatin, leptin, adiponectin and resistin.

**Methods:**

Peripheral blood mononuclear cells (PBMCs) were collected for 21 adult DM, 26 juvenile DM, 5 non-disease adult controls, and 6 non-disease pediatric controls at two time points: baseline and 6 months. Muscle biopsies from 5 adult DM patients and 5 non-disease adult controls were collected at baseline. Similarly, muscle biopsies from 7 juvenile DM patients and 5 non-disease pediatric controls were collected at baseline. The gene expression levels of leptin, adiponectin, resistin, visfatin and related inflammatory cytokines, IL-6, TNF- α, and housekeeping genes GAPDH, B2M, and ACTB were generated using a custom RT^2^ Profiler PCR Array.

**Results:**

Visfatin gene expression levels in peripheral blood were significantly higher in newly diagnosed adult DM cases compared to non-disease controls (*P* = 0.004) and these levels correlated with baseline clinical parameters such as age (r = 0.34, *P* = 0.020), male sex (r = −0.35, *P* = 0.017), prednisone use (r = −0.42, *P* = 0.006), and DMARD use (r = 0.35, *P* = 0.025). No significant association was found between change in visfatin gene expression levels and change in disease activity measures. While visfatin gene expression was significantly up-regulated in muscle tissue of juvenile DM patients (*P* = 0.028), in adult DM patients only a trend towards significance was observed (*P* = 0.08). Also, muscle gene expression levels of resistin were significantly elevated in both adult and juvenile DM patients compared respectively to non-disease adult and pediatric controls. Furthermore, an association between peripheral blood resistin gene expression and DM disease activity, including global, muscle, and extra-skeletal disease activity was also observed.

**Conclusion:**

Peripheral blood visfatin gene expression and muscle resistin gene expression are significantly increased in newly diagnosed adult DM patients. Further longitudinal studies should explore the possibility of using gene expression levels of adipokines such as visfatin and resistin as novel clinical diagnostic biomarkers in DM.

## Introduction

The idiopathic inflammatory myopathies (IIMs) are a heterogeneous group of chronic acquired disorders characterized by proximal muscle weakness and muscle inflammation [1,2]. In adults, dermatomyositis (DM) and polymyositis (PM) are common IIM conditions; in children, juvenile DM is the most common IIM [3]. Adult and juvenile DM are both characterized clinically by proximal muscle weakness; muscle inflammation; and particular rashes in the face or other extensor surfaces [4]. While some overlap between disease characteristics exist, adult and juvenile DM are considered separate entities with variable features [5]. For example, adults with DM are at an increased risk of malignancy and are more likely to develop interstitial lung disease. Meanwhile juvenile DM is associated with increased vasculopathy and calcinosis.

Pharmacological therapy for DM consists of corticosteroids and disease-modifying antirheumatic drug (DMARDS) [6,7]. Some patients with DM respond poorly to conventional treatment and many experience morbidity from side effects [8]. Therefore, there is a need for safer and more effective treatment in these diseases. While the etiology of DM is still unknown, investigations into their pathogenesis have confirmed the involvement of many pro-inflammatory cytokines. Consequently, one potential therapeutic approach would be to target individual cytokines that contribute to the pathogenesis of DM [8]. Recently it has been revealed that peripheral blood mononuclear cells (PBMCs) and muscle tissue of DM patients exhibit an interferon (IFN) gene expression signature which correlated with disease activity [8,9]. Interferons are potent cytokines that function to prevent viral replication (IFN-α and IFN- β), and mediate Th1 immune responses (IFN-γ). Therefore, studies should assess other cytokine pathways and evaluate their utility for monitoring disease activity and therapeutic response in patients with DM.

Adipokines, cytokines produced mainly by adipocytes, have been implicated in the regulation of immune and inflammatory responses. In fact, it is currently widely accepted that adipokines produced and secreted by white adipose tissue (WAT) are responsible for the chronic inflammatory state of visceral obesity [10]. While adipocytes are the most abundant cells in WAT, other cell types such as macrophages are also present [11]. Similarities between adipocytes and macrophages are observed, and pre-adipocytes can differentiate into macrophages [12]. Furthermore, although lymphocytes are not a constituent of WAT, there is often a close physical proximity between lymphocytes and WAT. For example, lymph nodes are generally surrounded by pericapsular adipose tissue [13,14]. Therefore, studies now support the idea of cross-talk between lymphocytes and adjacent adipocytes through a variety of adipokines. To date, the most heavily studied adipokines have been leptin, adiponectin, resistin, and visfatin. These adipokines are considered to be primarily pro-inflammatory factors, except for adiponectin which has been described to have anti-inflammatory properties [15].

Adipokines have also been implicated in vascular homeostasis [16]. In fact, certain adipokines have been found to be deregulated in patients with cardiovascular diseases and hypertension [16]. While the mechanisms underlying these associations remain unclear, it is thought that the crosstalk between adipose tissue and blood vessels is vital to vascular homeostasis. Studies have demonstrated that perivascular adipose tissue can produce many adipokines that contribute to the regulation of vascular function and local inflammation [16]. These findings are relevant to DM since vasculopathic changes are important features of DM. Changes in the nailfold capillaries, including dilatation, occlusion, arboreal loops, hemorrhages and vessel drop-out are well documented in DM, especially in juvenile DM [17].

The association between visceral obesity and chronic inflammation introduced the idea that adipokines may contribute to the pathogenesis of inflammatory-autoimmune conditions such as rheumatoid arthritis (RA) and systemic lupus erythematosus (SLE). While there are conflicting reports, most studies have shown increased serum levels of leptin, adiponectin, and resistin in both RA and SLE [18-26]. In the case of DM, the role of adipokines in the modulation of immune response has not been studied as extensively. Currently, only one report has evaluated serum resistin levels in DM [27]. In this study, Filkova et al. demonstrated elevated resistin levels in serum of patients with DM [27]. Thus, at present time most studies in inflammatory-autoimmune diseases have measured adipokine levels in serum, which may be unreliable to evaluate cytokine pathway activation [8,28]. Immunohistochemical studies for determining serum cytokine levels can be confounded by technical and biological limitations, including non-specific immunoreactivity, transient expression and low concentration [28]. Therefore, researchers have turned to PCR-based studies to examine cytokine gene expression in PBMCs (comprised of monocytes/macrophages, B and T lymphocytes, and natural killer cells) and have demonstrated that the real-time PCR technique is an alternative and potentially more precise way to evaluate cytokine pathways [8]. In fact, recent studies have documented the association of adipokines with metabolic syndrome at the gene expression level instead of the protein level [29].

The pathogenic role played by adipokines in inflammatory-autoimmune disorders such as juvenile DM and adult DM should be further explored at the gene expression level in a longitudinal study design. Therefore, we determined peripheral blood and skeletal muscle gene expression levels of leptin, adiponectin, resistin and visfatin in adult and pediatric patients with DM in comparison to non-disease controls at two different time-points and investigated their relation to clinical parameters and to disease activity measures.

## Materials and methods

### Patient characteristics

Blood and muscle biopsy samples were collected from pediatric and adult patients with newly diagnosed DM, sequentially recruited from clinics in the Mayo Clinic Division of Rheumatology, and Department of Medicine and Pediatrics and Adolescent Medicine (Rochester, MN). All patients met Bohan and Peter’s criteria for DM [30]. We collected PAXgene vacutainers for 21adult DM, 26 juvenile DM, and for adult (n = 5), and pediatric (n = 6) non-disease controls at two different time points: baseline and 6-month follow-up visit. Muscle biopsies for 5 adult DM patients from the same blood cohort were collected at baseline visit and 5 non-disease muscle biopsies served as controls. Similarly, muscle biopsies from 7 juvenile DM patients and 5 non-disease pediatric controls were collected. Disease activity was determined by the Myositis Disease Activity Assessment Visual Analogue Scales (MYOACT) Scoring System at time of blood draw at baseline and at the 6 month follow-up.

This study was carried out in accordance with research protocols approved by Institutional Review Boards of the Mayo Clinic. Patients and legal guardians signed informed consent and/or assent, and samples were de-identified in the laboratory.

### RNA isolation and quantification of adipokine gene expression

Total RNA was isolated from whole blood using PAXgene tubes with on-column DNase treatment according to manufacturer’s protocol (PreAnalytiX). Muscle was homogenized using a PowerGen 700 (Fisher Scientific) and RNA was isolated using Trizol (Invitrogen) and an RNeasy Mini Kit (Qiagen) with on-column DNase treatment. The mRNA gene expression levels of leptin, adiponectin, resistin, visfatin, IL6, TNFα, and housekeeping genes GAPDH, B2M, and ACTB were generated using a custom RT^2^ Profiler PCR Array run on an ABI 7900HT (Mayo Clinic Medical Genome Facility). Genes were normalized to the mean of three housekeeping genes and to experimental controls and were expressed as relative quantification.

### Statistical analysis

Data are expressed as the median (range), where indicated. A Wilcoxon rank sum test was performed for comparisons between two groups (e.g. adult DM versus adult controls; juvenile DM versus pediatric controls; and adult DM versus juvenile DM). A paired comparison test was performed to compare adipokine gene expression levels between baseline and 6 months. Spearman correlation coefficients were used to correlate any two variables. *P* values less than 0.05 were considered statistically significant. Analyses were performed using the SAS version 9.3 (SAS Institute Inc., Cary, NC, USA) statistical package.

## Results

### Baseline characteristics

The characteristics of patients included in the study are given in Table 1. Our study included 21 adult and 26 juvenile DM patients diagnosed by Bohan and Peter’s criteria for DM. The median (range) age (years) and gender were as follows: Juvenile DM 10.1(3.2-17.1) (38% male) and adult DM 53.0(20.0-79.0)(19% male). All adult DM and control subjects were Caucasian. Ethnicity in the juvenile DM group comprised; 77% Caucasian, 8% African American, 4% American Indian, 4% Pacific Islander, and 8% other. In terms of medication use at baseline, 70% of adult DM and 43% of juvenile DM cases were receiving prednisone or an equivalent, while 48% of adult DM and 33% of juvenile DM cases were receiving a DMARD. None of the DM patients had been diagnosed with malignancy.

**Table 1:** Baseline characteristics of patients with juvenile and adult dermatomyositis*

**Characteristic**	**JDM**	**DM**	**JDM and DM**
N	26	21	47
Age in years	10.1 (3.2-17.0)	53.0 (20.0-79.0)	16.7 (3.2-79.0)
Male sex (%)	10 (38%)	4 (19%)	14 (30%)
Time from Diagnosis date to baseline visit, years	0.05 (0–14.49)	0.17 (0–2.85)	0.10 (0–14.49)
Prednisone use (%)	9 (43%)	14 (70%)	23 (56%)
DMARD use (%)	7 (33%)	10 (48%)	17 (40%)
Global Disease Activity (0–100)	42.5 (2–81)	32 (3–80)	39 (2–81)
Muscle Disease Activity (0–100)	23.5 (0–83)	33 (0–83)	25 (0–83)
Global Extra-Skeletal Muscle Disease Activity (0–100)	35 (2–73)	32 (0–66)	33 (0–73)

*Values are presented as median (range) unless indicated otherwise. JDM = juvenile dermatomyositis; DM = dermatomyositis; N = number.

### Adipokine gene expression levels in peripheral blood

Gene expression levels of visfatin (*P* = 0.004) in peripheral blood were significantly elevated in adult DM patients when compared to non-disease adult controls (Table 2). Furthermore, gene expression levels of IL-6 displayed a non-statistically-significant trend toward lower levels in adult DM compared to non-disease adult controls (*P* = 0.06). Gene expression levels of TNF- α (*P* < 0.001) and adiponectin (*P* = 0.014) in peripheral blood were significantly lower in pediatric DM patients when compared to non-disease pediatric controls (Table 3). While gene expression levels of visfatin were lower in pediatric DM compared to non-disease pediatric controls, the difference did not reach significance (*P* = 0.05). Gene expressions levels of visfatin were increased in peripheral blood of adult DM when compared to juvenile DM (*P* = 0.017). Also, gene expressions levels of IL-6 (*P* < 0.001) and leptin (*P* < 0.001) were lower in peripheral blood of adult DM when compared to juvenile DM.

**Table 2:** Differences in peripheral blood and muscle gene expression levels of adipokines between adult dermatomyositis patients and non-disease adult controls*

	**Peripheral blood**			**Muscle**		
	**Adult DM**	**Non-disease adult controls**	***P*** **Value**	**Adult DM**	**Non-disease adult controls**	***P*** **Value**
	**(N = 21)**	**(N = 5)**		**(N = 5)**	**(N = 5)**	
Leptin	0.7 (0.6, 0.8)	0.8 (0.7, 0.8)	0.49	1.5 (0.9, 2.5)	1.4 (0.5, 1.9)	0.60
Adiponectin	0.1 (0.1, 12.1)	0.2 (0.0, 12.4)	0.63	1.7 (0.9, 4.6)	1.3 (0.8, 2.0)	0.60
Resistin	1.4 (0.9, 2.8)	0.6 (0.5, 2.5)	0.18	23.2 (7.7, 40.4)	2.6 (2.5, 7.4)	0.047
Visfatin	1.9 (1.7, 2.5)	0.3 (0.2, 1.1)	0.004	1.9 (1.8, 2.7)	1.0 (1.0, 1.1)	0.08
IL-6	0.4 (0.3, 0.7)	1.4 (0.7, 1.6)	0.06	2.9 (1.7, 4.0)	1.0 (0.8, 1.1)	0.047
TNF-α	0.7 (0.5, 0.8)	0.8 (0.7, 1.5)	0.28	1.8 (1.1, 2.4)	1.0 (0.9, 1.2)	0.25

*Values are presented as median (25^th^ percentile, 75^th^ percentile). Gene expression levels of adipokines were normalized to the mean of age-matched controls. *P*-value is from Wilcoxon rank sum test.

**Table 3:** Differences in peripheral blood and muscle gene expression levels of adipokines between juvenile dermatomyositis patients and non-disease pediatric controls*

	**Peripheral blood**			**Muscle**		
	**JDM (N = 26)**	**Non-disease pediatric controls (N = 6)**	***P*** **Value**	**JDM (N = 7)**	**Non-disease pediatric controls (N = 5)**	***P*** **Value**
Leptin	1.2 (1.0, 1.6)	1.3 (0.7, 2.8)	0.96	0.6 (0.3, 1.3)	1.4 (0.5, 1.9)	0.37
Adiponectin	0.1 (0.1, 0.2)	1.2 (0.1, 4.0)	0.014	2.6 (0.4, 7.7)	1.3 (0.8, 2.0)	0.46
Resistin	0.9 (0.6, 1.5)	1.0 (0.7, 1.5)	0.60	15.9 (10.5, 18.4)	2.6 (2.5, 7.4)	0.012
Visfatin	1.4 (1.0, 2.1)	2.1 (1.7, 2.5)	0.05	2.0 (1.4, 3.0)	1.0 (1.0, 1.1)	0.028
IL-6	1.2 (0.8, 1.5)	1.2 (0.6, 1.6)	0.92	1.6 (0.7, 1.7)	1.0 (0.8, 1.1)	0.22
TNF-α	0.6 (0.4, 0.7)	1.0 (0.9, 1.4)	<0.001	2.2 (1.1, 3.0)	1.0 (0.9, 1.2)	0.09

*Values are presented as median (25^th^ percentile, 75^th^ percentile). Gene expression levels of adipokines were normalized to the mean of age-matched controls. *P*-value is from Wilcoxon rank sum test.

### Adipokine gene expression levels in muscle tissue

Muscle gene expression levels of IL-6 (*P* = 0.047) and resistin (*P* = 0.047) were significantly higher in adult DM patients when compared to non-disease controls (Table 2). While muscle gene expression levels of visfatin were higher in adult DM compared to non-disease adult controls, the difference failed to reach significance (*P* = 0.08). Muscle gene expression levels of visfatin (*P* = 0.028) and resistin (*P* = 0.012) were significantly higher in pediatric DM patients compared to non-disease pediatric controls (Table 3). While gene expression levels of IL-6 in muscle were higher in adult DM compared to pediatric DM, the difference did not reach significance (*P* = 0.09).

### Correlation between baseline adipokine gene expression levels in peripheral blood and baseline clinical parameters

Baseline blood cell gene expression levels of IL-6 correlated with age (r = −0.64, *P* < 0.001) and prednisone use (r = 0.34, *P* = 0.03) (Table 4). Baseline blood cell gene expression levels of visfatin correlated with age (r = 0.34, *P* = 0.02), male sex (r = −0.35, *P* = 0.02), prednisone use (r = −0.42, *P* = 0.006), and DMARD use (r = 0.35, *P* = 0.02).

**Table 4:** Correlations between baseline peripheral blood gene expression levels of adipokines, baseline clinical parameters, and baseline disease activity measures*

	**Age**	**Male sex**	**Prednisone use**	**DMARD use**	**Baseline global extra-skeletal muscle disease activity**	**Baseline muscle disease activity**	**Baseline global disease activity**
Leptin	−0.74	0.01	0.23	0.10	0.04	−0.27	−0.06
	*P* < 0.001	*P* = 0.93	*P* = 0.14	*P* = 0.54	*P* = 0.81	*P* = 0.07	*P* = 0.68
Adiponectin	0.07	−0.07	−0.26	0.21	−0.15	−0.01	−0.06
	*P* = 0.62	*P* = 0.63	*P* = 0.10	*P* = 0.18	*P* = 0.31	*P* = 0.95	*P* = 0.70
Resistin	0.25	0.12	−0.34	−0.01	0.29	0.50	0.50
	*P* = 0.09	*P* = 0.44	*P* = 0.027	*P* = 0.95	*P* = 0.046	*P* < 0.001	*P* < 0.001
Visfatin	0.34	−0.35	−0.42	0.35	−0.20	−0.019	−0.11
	*P* = 0.020	*P* = 0.017	*P* = 0.006	*P* = 0.025	*P* = 0.17	*P* = 0.90	*P* = 0.46
IL-6	−0.64	0.03	0.34	−0.07	−0.03	−0.30	−0.14
	*P* < 0.001	*P* = 0.85	*P* = 0.029	*P* = 0.66	*P* = 0.87	*P* = 0.042	*P* = 0.34
TNF-α	0.12	0.003	0.28	0.22	−0.11	−0.25	−0.23
	*P* = 0.41	*P* = 0.98	*P* = 0.07	*P* = 0.16	*P* = 0.47	*P* = 0.09	*P* = 0.11

*Values are Spearman correlations, followed by *P*-values.

### Correlation between baseline adipokine gene expression levels in peripheral blood and baseline disease activity measures

Baseline blood cell gene expression levels of IL-6 correlated with muscle disease activity (r = −0.30, *P* = 0.04) (Table 4). Baseline blood cell gene expression levels of resistin correlated with global extra-skeletal muscle disease activity (r = 0.29, *P* = 0.046), muscle disease activity (r = 0.50, *P* < 0.001), and global disease activity (r = 0.51, *P* < 0.001).

### Change between baseline and 6 months in adipokine gene expression levels in peripheral blood

No significant differences (*P* > 0.05) were observed for the changes between baseline and 6 months in adipokine gene expression levels in peripheral blood of adult DM patients (Table 5). Meanwhile, in juvenile DM patients, only the change between baseline and 6 months in peripheral blood gene expression of IL-6 was significant (*P* =0.04) (Table 5).

**Table 5:** Change between baseline and 6 months in adipokine gene expression levels in peripheral blood of adult DM and JDM patients*

	**Adult DM**				**JDM**			
	**Baseline**	**6 months**	**Difference**	***P*** **-value**	**Baseline**	**6 months**	**Difference**	***P*** **-value**
Leptin	0.66 (0.25)	0.5 (0.22)	−0.16 (0.37)	0.26	1.14 (0.29)	0.81 (0.39)	−0.34 (0.54)	0.12
Adiponectin	11.8 (17.5)	3.2 (8.72)	−8.55 (17.25)	0.20	2.04 (5.23)	6.08 (16.6)	4.04 (17.82)	0.54
Resistin	3.08 (2.65)	2.84 (4.82)	−0.24 (4.7)	0.89	1.13 (0.82)	1.44 (1.98)	0.32 (2)	0.66
Visfatin	2.25 (1.01)	2.62 (1.5)	0.37 (1.43)	0.48	1.76 (0.87)	2.24 (1.55)	0.48 (1.77)	0.47
IL-6	0.48 (0.46)	0.42 (0.24)	−0.07 (0.42)	0.67	1.23 (0.73)	0.71 (0.3)	−0.52 (0.58)	0.04
TNF-α	0.61 (0.3)	0.67 (0.4)	0.06 (0.34)	0.65	0.6 (0.17)	0.63 (0.27)	0.03 (0.29)	0.76

*Values are presented as mean (SD). Gene expression levels of adipokines were normalized to the mean of age-matched controls. *P*-value is from paired comparison test.

### Correlation between change in adipokine gene expression levels in peripheral blood and change in disease activity measures

No significant associations (*P* > 0.05) between change from baseline to 6 months in adipokine gene expression levels in peripheral blood and change in disease activity measures were observed (Table 6). However, a non-statistically-significant trend was displayed for the association between change in gene expression levels of IL-6 in peripheral blood and change in global extra-skeletal muscle disease activity (r = 0.49, *P* = 0.05).

**Table 6:** Correlations between change in peripheral blood gene expression levels of adipokines and change in disease activity measures*

	**Change in global extra-skeletal muscle disease activity**	**Change in muscle disease activity**	**Change in global disease activity**
Leptin	−0.25	−0.11	−0.06
	*P* = 0.34	*P* = 0.70	*P* = 0.84
Adiponectin	0.16	−0.01	−0.05
	*P* = 0.56	*P* = 0.96	*P* = 0.85
Resistin	−0.23	0.42	0.14
	*P* = 0.39	*P* = 0.10	*P* = 0.61
Visfatin	−0.24	−0.24	−0.22
	*P* = 0.36	*P* = 0.37	*P* = 0.42
IL-6	0.49	−0.03	0.29
	*P* = 0.05	*P* = 0.90	*P* = 0.28
TNF-α	−0.38	0.01	−0.08
	*P* = 0.15	*P* = 0.97	*P* = 0.76

*Values are Spearman correlations, followed by *P*-values.

## Discussion

In this study, we investigated whether blood and muscle gene expression levels of certain adipokines are deregulated in adult and pediatric patients with DM. Our results indicate that genes of certain adipokines are significantly differentially expressed between adult DM and non-disease adult controls; and between juvenile DM and non-disease pediatric controls. Gene expression of visfatin is significantly up-regulated in peripheral blood of adult DM patients- compared to non-disease adult controls, while gene expression of TNF-α and adiponectin are down-regulated in peripheral blood of pediatric DM compared to non-disease pediatric controls. Additionally, our findings suggest that there are adipokine gene expression differences in adult DM versus juvenile DM in peripheral blood. Gene expression of visfatin was up-regulated, while IL-6 and leptin were down-regulated, in peripheral blood of adult DM when compared to juvenile DM.

Furthermore, we explored whether baseline gene expression levels of adipokines in peripheral blood associate with baseline clinical parameters. Interestingly, baseline gene expression of visfatin in peripheral blood, which is up-regulated in adult DM, correlated significantly with clinical parameters at baseline such as age, male sex, prednisone use, and DMARD use. Moreover, baseline peripheral blood resistin gene expression significantly correlated with DM disease activity, including global, muscle, and extra-skeletal disease activity. However, when exploring the predictive value of gene expression of adipokines in peripheral blood for change in disease activity measures by performing Spearman correlations, no significant associations were found.

Consistent with our findings of gene expression of adipokines in peripheral blood, our study also observed deregulation in gene expression of certain adipokines in muscle tissue. Gene expressions of IL-6 and resistin were significantly up-regulated in muscle tissue of adult DM patients compared to non-disease adult controls. Only a non-statistically-significant trend toward elevation of gene expression of visfatin in muscle tissue was observed in adult DM compared to non-disease controls. Moreover, gene expression of visfatin was significantly up-regulated in muscle tissue of pediatric DM patients compared to non-disease pediatric controls. Therefore, our over-all findings suggest the role of visfatin should be further explored in inflammatory-autoimmune disorders.

Visfatin, also known as pre-B-cell colony enhancing factor and nicotinamide phosphoribosyltransferase (NAMPT), is an adipokine produced predominantly by visceral fat that stimulates insulin signaling [18]. Serum levels of visfatin correlate with the amount of visceral fat and are increased in patients with obesity and type- 2 diabetes [31,32]. While visfatin is mainly produced by visceral adipose tissue, studies have demonstrated that it is also produced by several types of immune cells [33,34]. In fact, visfatin has been shown to have pro-inflammatory and immunomodulatory properties, and therefore is currently regarded as an important mediator of innate immunity and inflammation. Visfatin is synthesized in response to inflammation and up-regulates the production of pro-inflammatory and anti-inflammatory cytokines such as TNF-α, IL-6, IL-1β, IL-10 and IL-1Ra [35]. Also, visfatin promotes activation of T-cells by up-regulating the expression of co-stimulatory molecules such as CD40, CD54 and CD80 on monocytes [35]. There is evidence as well that visfatin serves as a potent chemotactic factor for monocytes and lymphocytes [35]. Lastly, visfatin, in conjunction to IL-7, can affect early B and T-lymphocyte development [35].

While reports on visfatin have focused heavily on its function on the metabolic syndrome and obesity, recently there have been reports on its association with inflammatory diseases. In both RA and SLE, serum levels of visfatin are increased compared to healthy controls [20,36]. Furthermore, in RA, serum levels of visfatin correlated with the degree of inflammation, severity of disease, and joint damage [18,36]. Moreover, inhibition of visfatin in a mouse model of RA showed reduced systemic and local inflammatory markers of arthritis, including reduced serum levels of IL-6, and a marked reduction in the number of Th17 cell, as well, as in the number and activation status of monocytes, macrophages, and neutrophils infiltrating arthritic joints [37]. Therefore, there is potential utility of targeting the visfatin gene for therapeutic intervention in arthritis [37].

In search of novel cytokine pathways that could potentially have utility for monitoring disease activity and therapeutic response in patients with DM, we explored the adipokine gene expression profile of adult and pediatric DM patients. To our knowledge, this is the first study to asses both peripheral blood and muscle gene expression levels of adipokines in patients with DM. While resistin had been reported to be increased in serum of DM patients by Filkova et al. [27], previous to our study, it was unknown whether adipokine gene expression levels in peripheral blood or muscle tissue were deregulated in DM. While peripheral blood gene expression levels of resistin were not found to be significantly different between DM patients and controls, we did observe that muscle gene expression levels of resistin were significantly elevated in both adult and juvenile DM patients compared respectively to non-disease adult and pediatric controls. Furthermore, our study showed an association between peripheral blood resistin gene expression and DM disease activity, including global, muscle, and extra-skeletal disease activity.

Resistin is a member of cysteine-rich proteins called “resistin-like molecules” or “found in inflammatory zone” [15,38]. While originally described as an adipocyte-specific hormone modulating insulin resistance in rodents, it is imperative to note that there are marked differences in the source of production of resistin between humans and rodents. In rodents, resistin is synthetized mainly by adipocytes; however in humans, it is chiefly synthesized in circulating monocytes and macrophages [15,38]. In humans, therefore, resistin is thought to regulate metabolic processes, adipogenesis, and inflammatory reactions. While the role of resistin in the pathology of muscle tissue in DM remains unknown, the association of resistin with muscle tissue inflammation in DM patients advocates further research in the area. Studies have demonstrated that resistin expression is up-regulated by pro-inflammatory cytokines, and in turn, resistin is able to induce the production of TNF-α, IL-6, IL-12, and IL-1β [15,39]. In fact, it was recently shown that resistin induces the production of these pro-inflammatory cytokines by acting as a competitive agonist for TLR-4, which is expressed in immune cells and immature muscle cell precursors [40]. These findings suggest that resistin may participate in muscle tissue damage by inducing pro-inflammatory cytokines through an interaction with TLR-4.

Because of the potential for transient expression and low concentration of cytokines, we opted to measure the adipokine gene profile in patients with DM at the gene expression level, and not the protein level. Previous studies have shown that gene expression profiling of PBMCs can be useful to identify distinct patterns of gene expression that distinguish patients with inflammatory-autoimmune disorders from non-disease controls [8,9,41]. Though our study looked at gene expression concentrations of adipokines in whole blood, instead of serum, we believe our results are in line with previous studies in autoimmune-inflammatory diseases demonstrating deregulation in serum adipokines. Nevertheless, a limitation of this study was the relatively small sample size. For this reason, our data must be interpreted carefully. Further studies should explore whether adipokines, such as visfatin and resistin, may serve as potential biomarkers for disease activity monitoring and therapeutic response in DM.

## Conclusion

Adipokines are mainly adipocyte-derived cytokines with pro-inflammatory and immunomodulatory properties in humans. Our study shows that gene expression levels of visfatin are elevated in peripheral blood of adult DM patients, and that these levels are associated with baseline clinical parameters such as age, sex, prednisone use and DMARD use. Also, we demonstrate that muscle gene expression levels of resistin are significantly elevated in both adult and juvenile DM patients compared respectively to non-disease adult and pediatric controls. Furthermore, an association between peripheral blood resistin gene expression and DM disease activity, including global, muscle, and extra-skeletal disease activity, was also seen. Nevertheless, the role of adipokines, such as visfatin and resistin, in the pathology of DM and their usefulness as biomarkers for disease activity monitoring remains to be elucidated.

## Abbreviations

DM: Dermatomyositis
PBMCs: Peripheral blood mononuclear cells
IIMs: Idiopathic inflammatory myopathies
PM: Polymyositis
DMARDS: Disease-modifying antirheumatic drugs
IFN: Interferon
WAT: White adipose tissue
RA: Rheumatoid arthritis
SLE: Systemic lupus erythematosus
MYOACT: Myositis Disease Activity Assessment Visual Analogue Scales
JDM: Juvenile dermatomyositis

## References

[1] Mastaglia FL, Garlepp MJ, Phillips BA, Zilko PJ (2003). Inflammatory myopathies: clinical, diagnostic and therapeutic aspects. Muscle Nerve.

[2] Hoogendijk JE, Amato AA, Lecky BR, Choy EH, Lundberg IE, Rose MR (2004). 119th ENMC international workshop: trial design in adult idiopathic inflammatory myopathies, with the exception of inclusion body myositis, 10–12 October 2003, Naarden, The Netherlands. Neuromuscul Disord.

[3] Rider LG, Miller FW (1997). Classification and treatment of the juvenile idiopathic inflammatory myopathies. Rheum Dis Clin North Am.

[4] Mammen AL (2010). Dermatomyositis and polymyositis: Clinical presentation, autoantibodies, and pathogenesis. Ann N Y Acad Sci.

[5] Robinson AB, Reed AM (2011). Clinical features, pathogenesis and treatment of juvenile and adult dermatomyositis. Nat Rev Rheumatol.

[6] Amato AA, Griggs RC (2003). Treatment of idiopathic inflammatory myopathies. Curr Opin Neurol.

[7] Aggarwal R, Oddis CV (2012). Therapeutic advances in myositis. Curr Opin Rheumatol.

[8] Greenberg SA, Higgs BW, Morehouse C, Walsh RJ, Kong SW, Brohawn P (2012). Relationship between disease activity and type 1 interferon- and other cytokine-inducible gene expression in blood in dermatomyositis and polymyositis. Genes Immun.

[9] Baechler EC, Bauer JW, Slattery CA, Ortmann WA, Espe KJ, Novitzke J (2007). An interferon signature in the peripheral blood of dermatomyositis patients is associated with disease activity. Mol Med.

[10] Fantuzzi G (2005). Adipose tissue, adipokines, and inflammation. J Allergy Clin Immunol.

[11] Curat CA, Miranville A, Sengenes C, Diehl M, Tonus C, Busse R (2004). From blood monocytes to adipose tissue-resident macrophages: induction of diapedesis by human mature adipocytes. Diabetes.

[12] Cousin B, Munoz O, Andre M, Fontanilles AM, Dani C, Cousin JL (1999). A role for preadipocytes as macrophage-like cells. Faseb J.

[13] Pond CM (2003). Paracrine interactions of mammalian adipose tissue. J Exp Zool A Comp Exp Biol.

[14] Pond CM (2003). Paracrine relationships between adipose and lymphoid tissues: implications for the mechanism of HIV-associated adipose redistribution syndrome. Trends Immunol.

[15] Krysiak R, Handzlik-Orlik G, Okopien B (2012). The role of adipokines in connective tissue diseases. Eur J Nutr.

[16] Even SE, Dulak-Lis MG, Touyz RM, Dinh Cat AN (2014). Crosstalk between adipose tissue and blood vessels in cardiometabolic syndrome: implication of steroid hormone receptors (MR/GR). Horm Mol Biol Clin Investig.

[17] Dugan EM, Huber AM, Miller FW, Rider LG (2009). Review of the classification and assessment of the cutaneous manifestations of the idiopathic inflammatory myopathies. Dermatol Online J.

[18] Gremese E, Tolusso B, Gigante MR, Ferraccioli G (2014). Obesity as a Risk and Severity Factor in Rheumatic Diseases (Autoimmune Chronic Inflammatory Diseases). Front Immunol.

[19] Chung CP, Long AG, Solus JF, Rho YH, Oeser A, Raggi P (2009). Adipocytokines in systemic lupus erythematosus: relationship to inflammation, insulin resistance and coronary atherosclerosis. Lupus.

[20] De Sanctis JB, Zabaleta M, Bianco NE, Garmendia JV, Rivas L (2009). Serum adipokine levels in patients with systemic lupus erythematosus. Autoimmunity.

[21] McMahon M, Skaggs BJ, Grossman JM, Sahakian L, Fitzgerald J, Wong WK (2014). A panel of biomarkers is associated with increased risk of the presence and progression of atherosclerosis in women with systemic lupus erythematosus. Arthritis Rheumatol.

[22] Vadacca M, Zardi EM, Margiotta D, Rigon A, Cacciapaglia F, Arcarese L (2013). Leptin, adiponectin and vascular stiffness parameters in women with systemic lupus erythematosus. Intern Emerg Med.

[23] Tanaka N, Kusunoki N, Kusunoki Y, Hasunuma T, Kawai S (2013). Resistin is associated with the inflammation process in patients with systemic autoimmune diseases undergoing glucocorticoid therapy: comparison with leptin and adiponectin. Mod Rheumatol.

[24] Ozgen M, Koca SS, Aksoy K, Dagli N, Ustundag B, Isik A (2011). Visfatin levels and intima-media thicknesses in rheumatic diseases. Clin Rheumatol.

[25] Scotece M, Conde J, Gomez R, Lopez V, Pino J, Gonzalez A (2012). Role of adipokines in atherosclerosis: interferences with cardiovascular complications in rheumatic diseases. Mediators Inflamm.

[26] Sglunda O, Mann H, Hulejová H, Kuklová M, Pecha O, Pleštilová L (2014). Decreased circulating visfatin is associated with improved disease activity in early rheumatoid arthritis: data from the PERAC cohort. PLoS One.

[27] Filkova M, Hulejova H, Kuncova K, Plestilova L, Cerezo LA, Mann H (2012). Resistin in idiopathic inflammatory myopathies. Arthritis Res Ther.

[28] Emslie-Smith AM, Arahata K, Engel AG (1989). Major histocompatibility complex class I antigen expression, immunolocalization of interferon subtypes, and T cell-mediated cytotoxicity in myopathies. Hum Pathol.

[29] Sadashiv, Tiwari S, Gupta V, Paul BN, Kumar S, Chandra A (2015). IL-6 gene expression in adipose tissue of postmenopausal women and its association with metabolic risk factors. Mol Cell Endocrinol.

[30] Bohan A, Peter JB (1975). Polymyositis and dermatomyositis (second of two parts). N Engl J Med.

[31] Sethi JK, Vidal-Puig A (2005). Visfatin: the missing link between intra-abdominal obesity and diabetes?. Trends Mol Med.

[32] Haider DG, Schindler K, Schaller G, Prager G, Wolzt M, Ludvik B (2006). Increased plasma visfatin concentrations in morbidly obese subjects are reduced after gastric banding. J Clin Endocrinol Metab.

[33] Brentano F, Schorr O, Ospelt C, Stanczyk J, Gay RE, Gay S (2007). Pre-B cell colony-enhancing factor/visfatin, a new marker of inflammation in rheumatoid arthritis with proinflammatory and matrix-degrading activities. Arthritis Rheum.

[34] Nowell MA, Richards PJ, Fielding CA, Ognjanovic S, Topley N, Williams AS (2006). Regulation of pre-B cell colony-enhancing factor by STAT-3-dependent interleukin-6 trans-signaling: implications in the pathogenesis of rheumatoid arthritis. Arthritis Rheum.

[35] Moschen AR, Kaser A, Enrich B, Mosheimer B, Theurl M, Niederegger H (2007). Visfatin, an adipocytokine with proinflammatory and immunomodulating properties. J Immunol.

[36] Sglunda O, Mann H, Hulejova H, Kuklova M, Pecha O, Plestilova L (2014). Decreased circulating visfatin is associated with improved disease activity in early rheumatoid arthritis: data from the PERAC cohort. PLoS One.

[37] Presumey J, Courties G, Louis-Plence P, Escriou V, Scherman D, Pers YM (2013). Nicotinamide phosphoribosyltransferase/visfatin expression by inflammatory monocytes mediates arthritis pathogenesis. Ann Rheum Dis.

[38] Toussirot E, Streit G, Wendling D (2007). The contribution of adipose tissue and adipokines to inflammation in joint diseases. Curr Med Chem.

[39] Bokarewa M, Nagaev I, Dahlberg L, Smith U, Tarkowski A (2005). Resistin, an adipokine with potent proinflammatory properties. J Immunol.

[40] Fu Q, Chen X, Cui H, Guo Y, Chen J, Shen N (2008). Association of elevated transcript levels of interferon-inducible chemokines with disease activity and organ damage in systemic lupus erythematosus patients. Arthritis Res Ther.

[41] Baechler EC, Batliwalla FM, Karypis G, Gaffney PM, Ortmann WA, Espe KJ (2003). Interferon-inducible gene expression signature in peripheral blood cells of patients with severe lupus. Proc Natl Acad Sci U S A.

